# A partition-based approach to identify gene-environment interactions in genome wide association studies

**DOI:** 10.1186/1753-6561-8-S1-S60

**Published:** 2014-06-17

**Authors:** Ruixue Fan, Chien-Hsun Huang, Inchi Hu, Haitian Wang, Tian Zheng, Shaw-Hwa Lo

**Affiliations:** 1 Department of Statistics, Columbia University, 1255 Amsterdam Avenue, 10th Floor, New York, NY 10027, USA; 2 ISOM, Hong Kong University of Science and Technology, Hong Kong; 3 Division of Biostatistics, School of Public Health and Primary Care, The Chinese University of Hong Kong, Hong Kong

## Abstract

It is believed that almost all common diseases are the consequence of complex interactions between genetic markers and environmental factors. However, few such interactions have been documented to date. Conventional statistical methods for detecting gene and environmental interactions are often based on the linear regression model, which assumes a linear interaction effect. In this study, we propose a nonparametric partition-based approach that is able to capture complex interaction patterns. We apply this method to the real data set of hypertension provided by Genetic Analysis Workshop 18. Compared with the linear regression model, the proposed approach is able to identify many additional variants with significant gene-environmental interaction effects. We further investigate one single-nucleotide polymorphism identified by our method and show that its gene-environmental interaction effect is, indeed, nonlinear. To adjust for the family dependence of phenotypes, we apply different permutation strategies and investigate their effects on the outcomes.

## Background

Genome-wide association studies (GWAS) have successfully discovered many common variants associated with complex diseases, but the single-nucleotide polymorphisms (SNPs) identified so far account for a small proportion of the total heritability in quantitative traits [1]. Increasing evidence shows that gene-environment (G×E) interactions are widely involved in the etiology of complex diseases, including diabetes, cancer, and psychiatric disorders [2,3]. The investigation of G×E interactions will not only facilitate the identification of novel genes whose marginal effects are undetectable, but also provide insights into disease etiology and hence greatly benefit drug development and personalized therapy.

The commonly applied methods to detect G×E interactions are based on linear or logistic regression models [4]. In particular, for quantitative outcomes, a linear model is considered in the form of

(1) $$y=\beta_{0}+\beta_{1}G+\beta_{2}E+\beta_{3}G\times E+\varepsilon$$

where *G *is the genotype of a SNP, *E *is the environmental factor, *ε *is a normally distributed random error, and $\beta_{3}$ is the coefficient corresponding to the interaction term. If $\beta_{3}=0,$ the conditional effect of the SNP is constant across different levels of the environmental factor and we conclude that there is no G×E interaction. This model assumes a linear interaction effect; given *G*, the outcome *y *is linearly related with *E*. However, in practice, it is likely that the interaction schemes are more complicated so that the linear model will probably fail to capture the interaction effect. Therefore, there is a pressing need to develop novel statistical approaches for genome-wide G×E interaction studies. Here we propose a nonparametric partition-based approach to detect G×E interactions and conduct a GWAS for hypertension using the real data set provided by Genetic Analysis Workshop 18 (GAW18). For each SNP, both the linear regression model and the proposed method are used to evaluate its interaction effect with each of the 4 environmental factors: age, gender, smoking status, and medicine. We note that, compared with the linear model, the proposed method is able to identify many additional SNPs. We further study the interaction pattern between SNP rs17206492 and medicine, and find that this interaction effect is, indeed, nonlinear. We also investigate different permutation strategies in the presence or absence of pedigree dependence of the phenotype.

## Methods

### Data set

The GAW18 data set consists of GWAS data and whole genome sequence data with longitudinal phenotypes for hypertension and related traits from Type 2 Diabetes Genetic Exploration by Next-generation sequencing in Ethnic Samples (T2D-GENES) Project 2. There are 939 individuals in total, and we include in our analysis only the 849 individuals with both phenotype data and imputed sequence information. Each individual has measurements for up to 4 time points. At each visit, systolic blood pressure (SBP) and diastolic blood pressure (DBP) were measured; covariates including age, use of antihypertensive medication, and current tobacco smoking status were also recorded. Gender and pedigree are known for each subject. Genotypes of odd-numbered chromosomes are provided. In our study, we focused on chromosome 3 as suggested by the workshop organizer for the sake of comparison. Although we had access to the answers for the simulated data set, we used only the real data set in our analysis.

### A general framework--a partition-based association measure

Suppose there are *n *independent subjects that can be separated by a partition *∏*. An association measure between the outcome *Y *and the partition *∏ *is defined as:

(2) $$I=\underset{\Pi_{i}}{\sum }\frac{n_{i}}{n}\frac{{\left(\bar{Y_{i}}-\bar{Y}\right)}^{2}}{{s_{y}}^{2}/n_{i}}$$

where $n_{i}$ is the number of subjects in partition *i*, ${\bar{Y}}_{i}$ is the average of the outcome *Y *for subjects in partition *i*, and $\bar{Y}$ and $s_{y}^{2}$ are the mean and variance of *Y *from all subjects. It has been shown that under the null hypothesis *∏ *does not have influence on *Y, I *asymptotically converges to a weighted sum of ${\chi^{2}}_{1}$ distributions [5]. It has higher power than linear regression or logistic regression models, even in sparse partitions.

### G×E association measure I

Consider a marker *G *and an environmental factor *E*. Suppose *G *has 3 phenotypes, AA, Aa, and aa (A refers to the major allele and a the minor allele), coded as 0, 1, and 2. Suppose *E *is divided into 3 categories: 0, 1, and 2. Hereby *G *and *E *together create 9 partitions for all subjects (Table 1). From the general framework in the last section, an association measure that evaluates the total effect of *G *and *E *on the phenotype is:

**Table 1:** Partitions created by genotypic and environmental factors

	*E = 0*	*E = 1*	*E = 2*	Total
*G = 0*	*n_00_*	*n_01_*	*n_02_*	*n_0_*
*G = 1*	*n_10_*	*n_11_*	*n_12_*	*n_1_*.
*G = 2*	*n_20_*	*n_21_*	*n_22_*	*n_2_*.
*Total*	*n._0_*	*n._1_*	*n._2_*	*n.*.

*n..*, Total number of subjects,*; n_ij_*, number of subjects in partition *ij, n_i_.*, number of subjects in group *G = i*; *n._j _*, number of subjects in group *E = j*.

(3) $$I_{T}=\overset{3}{\underset{i=1}{\sum }}\overset{3}{\underset{j=1}{\sum }}\frac{n_{ij}}{n..}\cdot \frac{{\left({\bar{y}}_{ij}-\bar{y}\right)}^{2}}{{s^{2}}_{y}^{}/n_{ij}}$$

where all the terms are similarly defined as before and *y *denotes the phenotype. The marginal effects of *G *and *E *can be obtained in a similar fashion:

(4) $$I_{G}=\overset{3}{\underset{i=1}{\sum }}\frac{n_{i\cdot }}{n..}\cdot \frac{{\left({\bar{y}}_{i∙}-\bar{y}\right)}^{2}}{{s^{2}}_{y}^{}/n_{i∙}};\;I_{E}=\overset{3}{\underset{j=1}{\sum }}\frac{n_{∙j}}{n..}\cdot \frac{{\left({\bar{y}}_{∙j}-\bar{y}\right)}^{2}}{{s^{2}}_{y}^{}/n_{∙j}}$$

The test statistic that measures the G×E interaction effect is defined as the difference between the total effect and the maximum of the two marginal effects:

(5) $$I_{G\times E}=I_{T}-max\left(I_{G},I_{E}\right)$$

The significance of *I_G×E _*is evaluated by the method of permutation.

### Permutation strategies

We consider 3 permutation strategies in our analysis: global permutation, local permutation, and residual permutation. Let *y_ij _*denote the phenotype of the *j^th ^*individual in the *i^th ^*pedigree. Global permutation is to permute phenotypes over all individuals. For local permutation, the phenotypes are permuted within each pedigree. In residual permutation, we first compute the residuals for each individual $e_{ij}=y_{ij}-{\bar{y}}_{i.}$, where ${\bar{y}}_{i.}$ is the average phenotype for pedigree *i*, then permute *e_ij _*over all subjects to obtain a permuted residual $e_{ij}^{*}$ for each individual. The permuted *Y *values $y_{ij}^{*}$ are obtained by $y_{ij}^{*}={\bar{y}}_{i.}+e_{ij}^{*}$. Both local permutation and residual permutation assume $y_{ij}={\bar{y}}_{i.}+\varepsilon_{ij}$, where $E\left(\varepsilon_{ij}\right)=0$ and $\left\{\varepsilon_{ij}\right\}$ are independent. Residual permutation further assumes that $\left\{\varepsilon_{ij}\right\}$ have the same distribution.

## Results

### Partitions created by environmental factors

The real data set from GAW18 contains the records of 4 environmental factors: age, gender, smoking status, and antihypertensive medication usage (medicine). Because gender is a binary variable, it partitions all individuals into 2 groups. Although this data set provides longitudinal measurements of age, smoking, and medicine, the records have many missing values (only 187 subjects have complete measurements for all 4 visits). Therefore, for each individual, we summarized these covariates by either the averaged value (for age) or the sum (for smoking and medicine) across different time points from available records and used these summarized quantities in our analysis. Similarly, averaged SBP and averaged DBP were considered as outcomes. Here we created 3 partitions by each of age, smoking, and medicine (Table 2).

**Table 2:** Partitions based on the summarized quantities of age, smoking status, or medicine

By age*	By smoking	By medicine
16~33.44 →Partition 0 33.45~50.30 →Partition 1 50.31~94.20 →Partition 2	0 → Partition 0 1 → Partition 1 2,3,4 → Partition 2	0 → Partition 0 1 → Partition 1 2,3,4 → Partition 2

^* ^The age group is divided by the 33% quantile (33.44) and 67% quantile (50.30). The minimum age is 16 and the maximum age is 94.2.

### SNPs with significant G×E interaction effects

**Figure 1:**
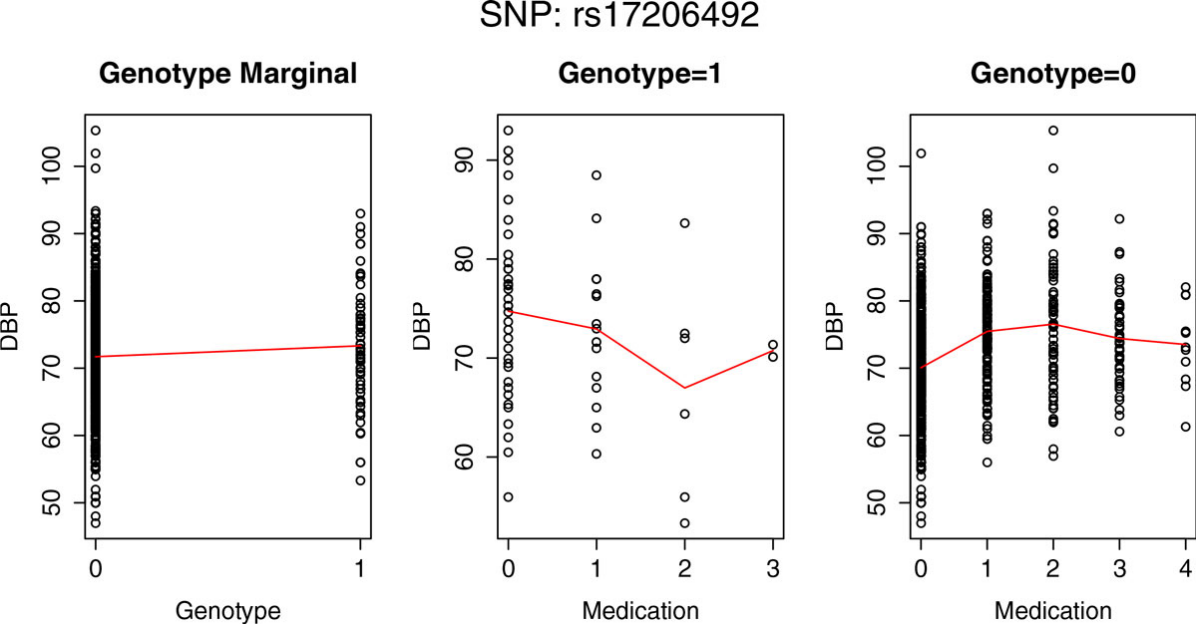
**G×E interaction effect of SNP *rs17206492 *and medicine**. The marginal effect of the genotype (*left*), the medication effect when genotype = 1 (*middle*), and the medication effect when genotype is 0 (*right*).

In the GWAS data set provided by GAW18, there are 62,915 SNPs on chromosome 3. For each SNP, we evaluated its interaction effect with each of the 4 environmental factors on both SBP and DBP using the linear regression model (LRM) and the proposed partition-based score *I (PBI). p *Values of LRM were derived from the asymptotic distribution of the regression coefficient $\beta_{3}$ and *p *values of *PBI *were computed from 10^7 ^permutations using global, local, or residual permutation procedures. Table 3 lists the number of SNPs with *p *values less than the Bonferroni-corrected significance level (7.9*10^−7^) for all interactions under consideration. Compared with LRM, *PBI *identified many additional significant SNPs, especially when testing the G×E interaction effects with medicine. The reason, we believe, is that the interaction modeled by LRM is restricted to the linear form, whereas *PBI *is able to capture nonlinear and complicated interaction patterns. To confirm our hypothesis, we further analyzed the SNP rs17206492, which was identified by *PBI *(using any of the 3 permutation strategies) to have strong G×Medicine interaction effect on DBP, but was not selected by LRM. The left panel of Figure 1 shows that the averaged values of DBP in individuals not carrying the minor allele (genotype 0) and in individuals carrying the minor allele (genotype 1) are almost the same, indicating that rs17206492 does not have strong marginal effect. However, with the increase of medication usage, when the genotype is 1 (middle panel of Figure 1), DBP first decreases and then increases; but when the genotype is 0 (right panel of Figure 1), DBP first increases and then decreases. This nonlinear interaction scheme cannot be detected by LRM, but is captured by our model-free test statistic *PBI*.

**Table 3:** Number of significant SNPs with *p *value less than 7.9*10^−^^7 ^*

Environmental factor	DBP				SBP			
	
	LRM	*PBI* *(GP)*	*PBI* *(LP)*	*PBI* *(RP)*	LRM	*PBI* *(GP)*	*PBI* *(LP)*	*PBI* *(RP)*
Age	0	4	7	3	6	16	33	20
Smoke	0	6	3	3	0	0	0	0
Gender	0	42	37	36	0	1	1	1
Medicine	4	80	53	33	1	65	65	57

*GP*, Global permutation; *LP*, local permutation; *LRM*, linear regression model; *PBI*, partition-based *I*; *RP*, residual permutation.

*7.9*10^−7 ^is the Bonferroni corrected *p *value.

### Effect of different permutation strategies

**Figure 2:**
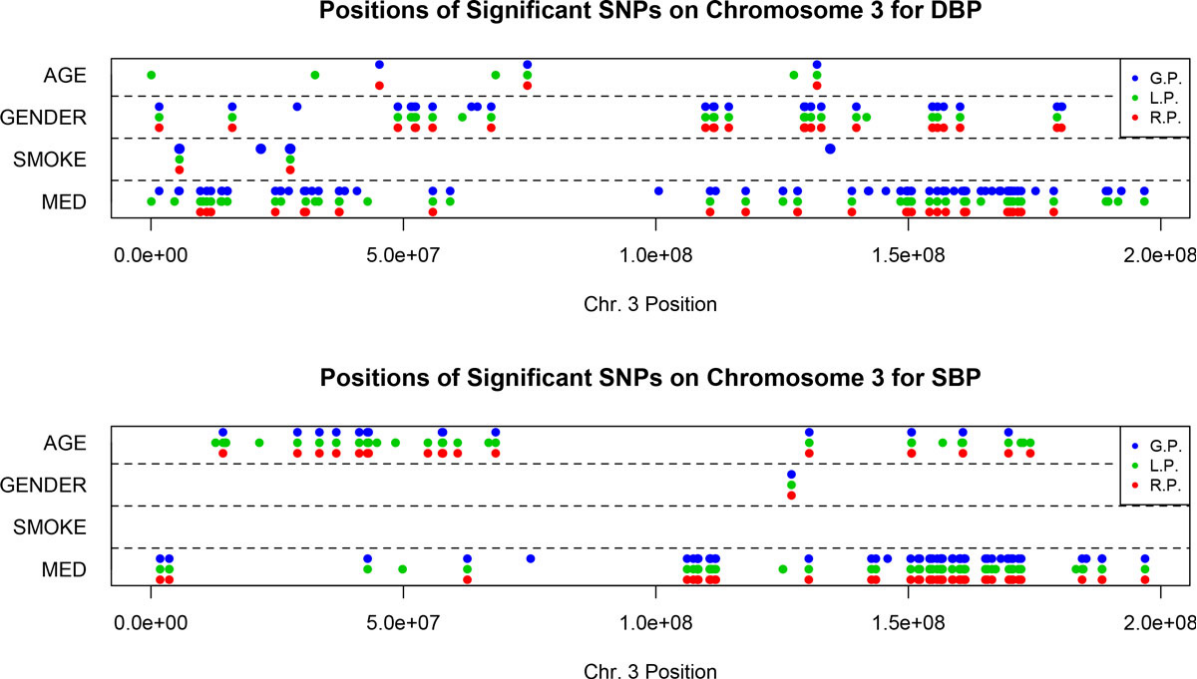
**Positions of SNPs identified to have significant G×E interaction effects by *PBI *using different permutation strategies for both SBP and DBP on chromosome 3**.

There are 20 pedigrees in the GAW18 data set. Both the analysis of variance (ANOVA) test and the nonparametric Kruskal-Wallis test indicate that the mean DBP values of different pedigrees are different, whereas the mean SBP values are the same (Table 4). When evaluating the *p *values of *PBI*, we performed 3 types of permutation: global (GP), local (LP), and residual (RP) permutations. Both LP and RP adjust for familial relatedness between individuals. For SBP, except for the environmental factor *age*, the results from 3 permutation methods coincide substantially (see Table 3 and Figure 2), which is consistent with the conclusion from ANOVA and Kruskal-Wallis test. In contrast, for DBP, the results of GP are quite different from the results of LP or RP, especially when assessing the interaction effect with medicine (see Table 3 and Figure 2). In this situation, the results from LP or RP are more reliable because they take into account the family dependence of the phenotype. In addition, LP tends to select more markers than RP; this may be because the data violate the assumption that $\left\{\varepsilon_{ij}\right\}$ have the same distribution. Moreover, SNPs identified by LP and RP overlap considerably and the consistency of results from these two permutation strategies can be an indicator of true signal.

**Table 4:** *p *Values for testing the pedigree dependence of SBP and DBP

	ANOVA test	Kruskal-Wallis test
SBP	0.155	0.433
DBP	0.000625	0.0004226

## Discussion

In this paper, we have proposed a partition-based approach *PBI *to detect G×E interactions, which is nonparametric and model-free. The test statistic is derived from a partition-based measure *I*, and the interaction information score *I_G×E _*is defined as the difference between the total score *I_T _*and the maximum of the marginal scores. Intuitively, if the genetic and the environmental factors have strong interaction effect, *I_T _*will be far greater than both marginal scores; hence *I_G×E _*will be positive and large. If not, *I_T _*will be no greater than at least 1 of the marginal scores. Therefore, *I_G×E _*evaluates the amount of influence of the G×E interactions on the phenotype.

When applied to the real data set about hypertension provided by GAW18, *PBI *identified many more markers than the traditional linear regression method. Because our approach is model-free, it is able to capture complicated interaction patterns that are difficult to detect in linear model. The significance of *I_G×E _*is evaluated by permutation. LP and RP adjust effectively for the family dependence of the phenotype. Despite the fact that the proposed procedure selects more SNPs than linear regression, there is very little experimental evidence of G×E interactions for hypertension in the current literature to verify our findings. Therefore, biological studies will be required to investigate our results. Modifications of *PBI *have successfully identified gene-gene interactions and constructed genetic networks for breast cancer [6] and rheumatoid arthritis [7]. Moreover, *PBI *can be extended to evaluate the interaction effects between rare variants and environmental factors. Because of the low frequencies of rare variants (<1%), we can apply a gene-based approach by collapsing rare variants in a gene [8-11] and creating partitions based on the collapsed information.

## Competing interests

The authors declare that they have no competing interests.

## Authors' contributions

SHL and RF designed the study. RF, CHH and SHL performed the study. RF, CHH, IH, HW, TZ and SHL contributed to analysis of the data. RF and SHL drafted the manuscript. All authors read and approved the final manuscript.
